# A Systematic Review of Evidence-Based High School Melanoma Prevention Curricula

**DOI:** 10.1007/s13187-023-02294-9

**Published:** 2023-04-12

**Authors:** Gina N. Calco, Victoria E. Orfaly, Carter K. Haag, Andrew Hamilton, Elizabeth Stoos, Sancy A. Leachman

**Affiliations:** 1 Oregon Health & Science University School of Medicine, Portland, OR 97239 USA; 2 Department of Dermatology, Oregon Health & Science University, 3303 SW Bond Ave, CH16D, Portland, OR 97239 USA; 3 Oregon Health & Science University Library, Oregon Health & Science University, Portland, OR 97239 USA

**Keywords:** Medical education, Melanoma, Sun protection, UV damage, Public health, Skin cancer

## Abstract

Incorporation of melanoma prevention behaviors into daily lifestyles is difficult. Data suggest that high school educational programs on skin cancer prevention can be successful and should incorporate evidence-based teaching and learning strategies to achieve greatest impact. The goal of this systematic review is to describe evidence-based educational practices for a high-school melanoma curriculum through a comprehensive review of the literature. Ovid MEDLINE, Embase, CINAHL, and PyscINFO were searched in June 2020 for all original articles published between June 18, 1946 and June 17, 2020. All studies that used an educational curriculum to promote sun safety, skin exams, and early detection to high school students were included. A total of 25 studies with 22,683 adolescent participants were analyzed. Sixteen studies showed a significant increase in knowledge, twenty-one studies showed changes in behavior, and fifteen studies showed significant changes in attitudes. Limitations of this review include the heterogeneity of implementation and outcome reporting of educational curricula. These findings support incorporating active learning strategies as key aspects of creating an effective curriculum aimed at the prevention and early detection of melanoma.

## Introduction

Prevention and early detection of melanoma has the potential to decrease lifetime risk and increase survivorship [1], but formal public health education efforts have been limited. This public health education need goes well beyond education of sun safety facts-and-figures, requiring stimulation of behavioral change to show impact. Despite evidence that performing a behavior at a younger age increases likelihood of continuing that behavior as an adult [2], a high-school educational solution that motivates positive behavioral change can be difficult to achieve and maintain.

School-based skin cancer curriculums employ evidence-based educational strategies (educational theories) to increase knowledge and promote behavioral change, which are the outcomes used to assess quality and efficacy. Incorporation of educational theories into curriculums provide a framework for effective and replicable teaching and assessment of motivations behind behavior change [3]. However, not all disciplines may realize or utilize this valuable knowledge when designing interventions. The goal of this review is to characterize the use of evidence-based learning theories in high school curricula for melanoma prevention. We have specifically examined published data from adolescent curricula that incorporates elements shown to improve knowledge, attitudes, and behaviors in the prevention and detection of melanoma.

## Methods

### Data Sources and Search Strategies

A systematic review was conducted on June 18, 2020 to identify studies that utilized an educational curriculum to teach high school-aged adolescents about the prevention and early detection of melanoma. Search strategies for this study were designed and conducted by a senior health sciences librarian (A.H.) with input from investigators (V.E.O. and G.N.C.) and were performed in Ovid MEDLINE, Embase, CINAHL, and PyscINFO. Results of the searches were limited to articles published or translated into the English language. This review adhered to Preferred Reporting Items for Systematic Reviews and Meta-analyses (PRISMA) reporting guidelines, which outlines exclusion criteria available via Mendeley at https://data.mendeley.com/datasets/ywv2kdgfcv/1. Search terms used in the strategies included concepts related to early detection, screening, skin exams, skin neoplasms, school health services, patient education, health education, preventative health services, attitudes, behavior, sunlight, sunburn, ultraviolet rays, high school, secondary school, and melanoma. Detailed search strategies are available via Mendeley at https://data.mendeley.com/datasets/ywv2kdgfcv.

### Study Selection

A total of one thousand, one hundred and sixty-nine papers were identified. We reviewed all titles and abstracts and included original research studies published between June 18, 1946 and June 17, 2020. Only studies involving a skin health-based curriculum delivered in schools with high school-aged adolescents (grades 9–12) as the target audience were included. We excluded eight studies that did not include high school-aged adolescents. Other exclusion criteria were community-based programs not conducted in a school setting, guidelines, policies, and studies without detailed curriculum information (supplemental figure 1 available via Mendeley at https://data.mendeley.com/datasets/ywv2kdgfcv). We ultimately selected 41 papers for full-text analysis and review, which identified sixteen papers that did not meet inclusion criteria and were excluded. Twenty-five articles were retained for final analyses.

### Curriculum Evaluation and Design

School curricula were evaluated on curriculum format, length, designated instructor, if interactive learning elements were included, and whether curriculum design was based on an educational theory and if so, which theory. We looked for whether the curriculum described significant improvements in knowledge, attitudes, behavior, confidence in abilities, and early detection compared to control groups or participants who did not receive the curriculum.

## Results

A total of twenty-five publications were analyzed (Table 1) with a cumulative 22,683 adolescent participants. Thirteen of the curricula were designed for schools across the USA [5, 7, 10–13, 16, 17, 21–24, 28], and the other twelve were implemented across Australia [14, 25–27], Europe [9, 15, 18–20], South America [6], and Asia [4, 8]. Eleven of the analyzed studies were randomized controlled trials [6, 9, 11, 12, 14, 15, 19, 23, 25–27], while one was described as a non-randomized controlled trial [18]. Other studies incorporated prospective cohorts [5, 7, 10, 16, 17, 21, 22, 24, 28], cross-sectional [20], or quasi-experimental [4, 8] designs. Although the randomized controlled trial by Kouzes et al. was included in our final analysis, we only examined the high school portion of the cohort to keep within our selection criteria.

**Table 1:** Characteristics of the twenty-five studies included in final analysis

Study	Study design	Quality of evidence	Number of participants
Khani et al. [4], 2019	Quasi-experimental	2	300
Irwin et al. [5], 2007	Prospective cohort	2	1214
Brinker et al. [6], 2018	RCT	1	1260
Geller et al. [7], 2005	Prospective cohort	2	344
Sümen et al. [8], 2015	Quasi-experimental	2	567
Hughes et al. [9], 1993	RCT	1	543
Swindler et al. [10], 2007	Prospective cohort	2	517
Jia et al. [11], 2020	RCT	1	271
Tuong et al. [12], 2015	RCT	1	50
Wu et al. [13], 2019	Controlled trial	2	1573
Hawkes et al. [14], 2012	RCT	1	400
Miljković et al. [15], 2014	RCT	1	5360
Kamell et al. [16], 2011	Prospective cohort	2	1260
Kouzes et al. [17], 2017	Prospective cohort	2	100*
Kristjánsson et al. [18], 2003	Controlled trial	2	184
Aarestrup et al. [19], 2014	RCT	1	2323
Brinker et al. [20], 2017	Cross-sectional	4	205
Cassel et al. [21], 2018	Prospective cohort	2	208
Loescher et al. [22], 2019	Prospective cohort	2	220
Katz et al. [23], 1991	RCT	1	251
Davis et al. [24], 2015	Prospective cohort	2	1284
White et al. [25], 2019	RCT	1	382
White et al. [26], 2010	RCT	1	80
Lowe et al. [27], 1999	RCT	1	3400
Kamin et al. [28], 1993	Prospective cohort	2	387

Quality of evidence scale based on the JAMA Dermatology modified Oxford Centre for Evidence-based Medicine for ratings of individual studies [29]. Evidence is ranked as follows: (1—RCT), (2—Well-designed controlled trial without randomization; prospective comparative cohort trial), (3—Case-control studies; retrospective cohort study), (4—Case series with or without intervention; cross-sectional study), (5—Opinion of respected authorities; case reports)

*RCT* randomized control trial

*Only high school students included in count

Eleven studies used a lecture to deliver curriculum content [4, 5, 10, 13, 15, 17, 18, 21–24, 28], while ten studies incorporated a video [4, 8, 9, 12, 14, 18, 19, 24, 27, 28] (Table 2). Although not all studies reported the length of the curriculum, of those that did, the average duration was 106 min. Curricula ranged from 5 min [12] to 7 h [7]. Nearly half of the studies used a curriculum that took an hour or less [5, 6, 8, 10, 12, 16, 18, 20–24]. While the majority of curricula used school teachers [5, 7, 9, 17–19, 21, 27, 28] to instruct the class, a few studies used medical students [6, 16], university students [24], or high school peers [22]. Eighteen of the twenty-five studies mentioned incorporating an interactive element into their sun safety curriculum. The most commonly included interactive elements were games or activities [5, 11, 20, 25, 27, 28], such as a “shopping spree” game in which participants were asked to select a sun-protection and/or acne-prevention product based on ingredients and labeling [5]. Other examples of activities included creating a campaign to encourage sun safety, role playing, debates, advocacy on social media, and UV skin analyzer activities. Some of the studies also based their educational programs in different types of educational theories.

**Table 2:** Educational curriculum characteristics (*n* = 25)

Characteristic*	No. of studies (%)	Featured in our curriculum?	Study
Medium used (> 1 possible)			
Lecture	11 (44)	Yes	[4, 5, 10, 13, 15, 17, 18, 21–24, 28]
Video	10 (40)	Yes	[4, 8, 9, 12, 14, 18, 19, 24, 27, 28]
Workbook/pamphlet/worksheet	3 (12)	Yes	[9, 11, 13]
Online module	3(12)	Yes	[6, 20, 22]
Length			
1 h or less	12 (48)	Yes	[5, 6, 8, 10, 12, 16, 18, 20–24]
Greater than 1 h	7 (28)	No	[7, 14, 15, 25–28]
Not reported	6 (24)	-	[4, 9, 11, 13, 17, 19]
Designated instructor			
School teachers	9 (36)	Yes	[5, 7, 9, 17–19, 21, 27, 28]
Medical students	2 (8)	Yes^†^	[6, 16]
University students	1 (4)	Yes	[24]
High school peers	1 (4)	No	[22]
Curriculum interactive elements (> 1 possible)			
Games/activities	6 (24)	Yes	[5, 11, 20, 25, 27, 28]
Role play	2 (8)	No	[25, 27]
Discussion	8 (32)	Yes	[11, 12, 14, 17, 18, 21–23, 30]
Image identification	1 (4)	Yes	[11]
Writing/drawing	5 (20)	No	[14, 18, 19, 25, 27]
Technology/photographing	6 (24)	No	[6, 13, 14, 20, 22, 24]
Goal setting	3 (12)	Yes	[13, 14, 25]
Educational theory			
Theory of planned behavior	4 (16)	Yes	[6, 14, 25, 26]
Appearance-based focus	3 (12)	Yes	[9, 15, 20]
Gamification	3 (12)	Yes	[5, 11, 22]
Goal setting	3 (12)	Yes	[13, 14, 25]

% = percentage of studies that had each named element

*Characteristics are not mutually exclusive and more than one may be present in any one curriculum

^†^Medical students instruct university students in a “train-the-trainer” program design

The Theory of Planned Behavior was the most commonly implemented educational theory among the studies we reviewed [6, 14, 25, 26]. The curricula that implemented this theory reported outcomes showing significant improvement in sun protective behaviors [25, 26] as well as effecting positive attitude changes associated with the behaviors of interest [26]. The next most commonly utilized teaching strategies incorporated an appearance-focus for adolescents [9, 15, 20], gamification theory [5, 11, 22], and goal setting [13, 14, 25]. Only one study of the three studies that used an appearance-based focus reported a significant change in some, but not all, skin cancer prevention behaviors [15]. In contrast, all studies that reported incorporating gamification theory [6, 14, 25, 26] or goal setting [13, 14, 25] activities and measured associated outcomes found significant increases in student knowledge gain or behavioral change.

Most studies measured changes in knowledge [4, 5, 7–11, 16–18, 22–24, 27, 28], behavior [4–10, 13–22, 24–27], or attitudes [4, 8–10, 14, 17–19, 22, 24–27] of participants receiving the curriculum. Knowledge questions were assessed via true/false, yes/no, or multiple-choice questions. Surveys were frequently given directly after curricula and follow-up occurred most often after 1 month of study. Three studies reported 6-month follow-up [7, 15, 19], and one reported 12-month follow-up [21]. Of the studies that report this longer follow-up, one showed a significant increase in knowledge at 6 months [7], and the other showed an increase in knowledge at 12 months relative to baseline [21]. The other two showed increases in some, but not all measured sun protective behaviors [15, 19]. While many of the behavior questions were based on adolescent reported or intended behaviors, one study [21] used direct field observation in their assessment of student behavioral changes. This study tested a school-based intervention for multiethnic high school students and successfully demonstrated the feasibility of objectively measuring students’ sun protection behavioral changes in a school population, although they did not report statistically significant changes in the observed behaviors. Sun safe and skin cancer preventative behaviors included the use of sun protective clothing (such as hats, long sleeves, and sunglasses), shade, tanning beds, and time spent outside. Attitude assessments focused on beliefs around tanning, peer perceptions, and personal appearance. Despite significant changes in 94% of studies that examined knowledge, significant changes in behavior (48%) and attitude (47%) were less frequently observed in the selected studies (Table 3).

**Table 3:** Educational curriculum outcome measurements

Outcome	No. of studies (%) (*n* = 25)	No. of studies with significant increase in outcome (%)
Knowledge	16 (64)	15 (94)
Behavior	21 (84)	10 (48)
Attitude	15 (60)	7 (47)
Confidence	3 (12)	1 (33)
Early detection	7 (28)	1 (14)

Only three studies examined student confidence, and only one of these demonstrated improvement in confidence. Students in one of these studies did not exhibit greater confidence in their ability to identify worrisome lesions after the curriculum [11]. Another study, however, showed an increase in self-confidence and the belief in their ability to perform sun safe behaviors [4]. The final study examined confidence in participant’s knowledge on skin cancer [23], which was significantly higher among students taught the curriculum. Interestingly, confidence ratings in the group that received the curriculum remained high despite a decrease in their knowledge as measured by test performance.

Seven [6, 7, 11, 13, 16, 22, 23] of the twenty-five schools in our review included early detection as part of their content, primarily in the form of teaching the ABCDE rules (Asymmetry, Border, Color, Diameter, Evolving) [31]. Of these studies only one reported a significant increase in the ability to define the five rules of early detection of skin cancer [7]. While five of the studies that included early detection found a significant change in general knowledge [7, 11, 16, 22, 23], only three reported changes in intended behaviors [13, 16, 22]. Two of these three studies used a 50-min lesson [16, 22], and all three incorporated some kind of interactive element into their curriculum. Although none of the studies that reviewed early detection reported on attitude changes, two found an increase in confidence [11, 23] in performing self-skin checks and identifying worrisome lesions.

## Discussion

There is a wide array of evidence-based educational theories that have promising effects in creating behavior change, especially in the health education setting [32]. The Theory of Planned Behavior (TPB) is based on conceptualizing a behavior as the result of behavioral intention, attitude, social norms, and perceived ability to perform that behavior (perceived behavioral control) [33]. This theory has been shown to positively change behavioral control and attitudes in health-related behaviors [34]. The utilization of game elements to enhance motivation and engagement in learning tasks, also known as gamification [10, 11], has been shown along with intentional goal setting be a critical component in health behavior changes [12, 35–38]. For example, incorporating specific and challenging goals produces performance improvement [32]. The Cognitive Theory of Multimedia Learning is a framework designed to build on cognitive processes to enhance learning and has also demonstrated improvement in both short and long-term retention and sustained application of knowledge in medical education [30, 38]. Lastly with regard to skin cancer education focus, discussing effects of UV radiation on premature aging has been shown to have a specific motivational effect on the adolescent population to practice sun safe behaviors [39].

In addition to what is seen in the education literature, our review showed that across multiple theories (TPB, gamification, and goal setting) there was a consistent trend of significant improvement in either student knowledge or behavioral change when these design elements were incorporated. However, despite this success both within the general and skin cancer education realms, our review also showed that these evidence-based theories are not routinely incorporated into skin cancer prevention curricula. We found that no single element appears to be consistently used across all of the studies reviewed. This could be in part why many studies reported significant increases in knowledge gained after completing the curriculum, but less than half of those led to significant or sustained changes in behavior or attitude. We believe that with the investment of efforts upfront to design curriculums with these theories, we can better maximize the efforts that are already taken to implement these programs.

Of note, much of the educational literature demonstrating the effectiveness of learning theories has been done in adult learner populations. While literature on these theories amongst adolescents are limited, the studies in this review show that high school aged adolescents appear to have the same positive outcomes as adults when learning theories are implemented consistently and effectively. This is in contrast to younger children aged 5–13 years old, who likely absorb information differently and have substantial differences in processing and retaining information compared to adults [40]. Furthermore, the direct effects on behavioral change are potentially more difficult to ascertain in learners 5–13 years old in the immediate post-intervention period as they may have less agency to make decisions or engage in certain behaviors. Therefore, while behavior change is possible at earlier ages, the focus of this review did not include these younger children in elementary or middle school and focused on adolescents that are likely to respond to these methodologies.

Additionally, the studies represented in this review span education systems across the globe from Australia to South America. The differences in educational norms and styles between different countries may have impacted the findings of each individual study. It is plausible that, when implemented within the respective cultural context, these learning theories have the potential to reproduce the same positive behavioral and knowledge effects, regardless of their country of origin. Indeed, there are little data available to suggest that the effects of TBP, gamification, or goal-setting learning theories are differentially influenced when developed in the context of the local culture [41]. However, consistent implementation of these learning theories in health curricula on a global scale is necessary to fully assess for these differences.

Further observations made while reviewing the literature include the lack of early detection and involvement of family members in existing curriculum. Future curricula should expand on the previous studies by measuring early detection related knowledge and attitudes beyond the ABCDE rules and emphasizing self-skin exams, instructing when to see a health care provider, and promoting a discussion of individual and family risk factors through the various interactive games throughout the lesson. Many melanomas are found by friends and family members, but usually later in their development; thus, students could have the opportunity to impact early detection by taking home worksheets and sharing it with their family. Caregivers have significant influence on their children’s behavior through behaviors they model, such as self-skin exams and wearing sunscreen [42], as well as by provision of sunscreen or access to a healthcare provider. Skin cancer, like many public health problems, extends beyond the individual level and can best be addressed when we consistently try to incorporate families, communities, and larger social structures in our interventions.

The studies in this review shed light on the design elements that can and should be incorporated into skin cancer curricula. However, it also sheds a light on the paucity of rigorous studies that exist on a global scale with our review incorporating only forty-one studies since 1946. Even amongst the studies that do exist, only four incorporated any long-term follow-up which makes it difficult to establish best practices for long-term retention and behavioral change. We recognize that for some programs, long-term follow up is not always feasible to obtain. However, even fewer studies measured student confidence despite the correlation between increased confidence and behavioral change [43]. Although there is an interest in this work and efforts on a community level to teach adolescents about skin health and safety, rigorous prospective data will be necessary to evaluate these public health interventions and foster change.

Finally, this review highlights the importance of interdisciplinary collaboration in educational public health interventions. Knowledge about the effectiveness of educational intervention strategies and evidence-based pedagogy, beyond the scope of well-intended health care providers and public health professionals, should be sought. Recognizing this need and intentionally incorporating interdisciplinary expertise into thoughtful curriculum design can be a crucial stepping stone, maximizing the efforts already being undertaken in schools worldwide for any number of educational efforts.

## Limitations

Our review is limited by the heterogeneity of both implementation and outcome reporting of skin cancer educational curricula. Self-reporting surveys were used to assess outcomes of many of the studies, which differed substantially between each curriculum. While it would have been beneficial to compare the knowledge, attitude, and behavioral outcomes between melanoma curricula, this was not possible due to these substantial differences. Therefore, we relied on the assumption that educational theories reported by each curriculum was incorporated per the standards described in the educational literature. Furthermore, because of developmental differences between adolescents of different ages we only included those studies that used high school-aged adolescent participants limiting the expansion of our conclusions to younger or older adolescent populations.

## Conclusion

In summary, although several educational programs have targeted skin cancer prevention in an adolescent population, few incorporate multiple well-studied educational theories and early detection as key parts of their curricula. This leads to varied and inconsistent changes in skin cancer prevention and detection knowledge, behaviors, attitudes, and confidence. Incorporating activities based on the theory of planned behavior, gamification, and goal setting theory of motivation is important in creating a measurable curriculum aimed at the prevention and early detection of melanoma.

As with many public health efforts, design of a meaningful public health intervention requires the collaboration of experts across many different fields. Melanoma prevention and early detection efforts, in particular, rely heavily on initiatives set by dermatologists that are made with the community in mind. It is for this reason that we strongly advocate physicians to utilize the research done by the health education community when designing community or patient education materials.
